# Supplementary material for: Single-electron induced surface plasmons on a topological nanoparticle
Source: Nat Commun. 2016 Aug 5;7:12375. doi: 10.1038/ncomms12375 (PMC4980453; doi:10.1038/ncomms12375)
Supplement: Supplementary Information — Supplementary Figures 1-2, Supplementary Notes 1-3 and Supplementary References [file ncomms12375-s1.pdf]

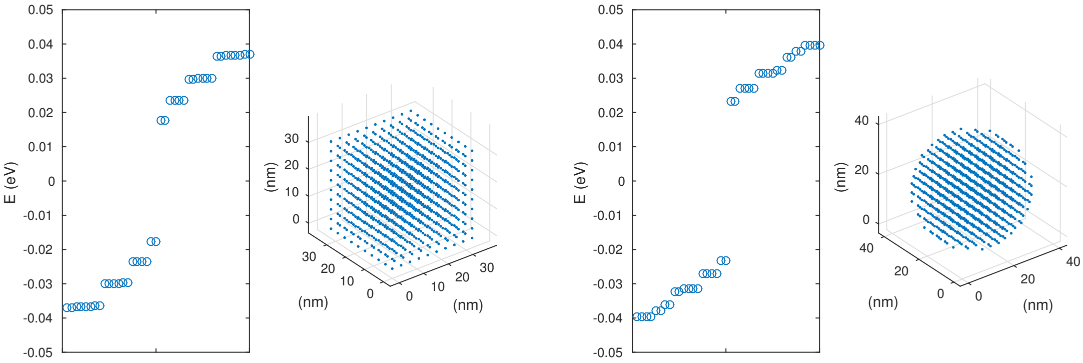

Supplementary Figure 1: Left: energy spectrum of a cube made of $N = 1000$ lattice points corresponding to $R = 22.3\,\mathrm{nm}$ in Fig. 2 of the article ($NL^3 = 4\pi R^3/3$) Right: Same for a sphere made of $N = 895$ lattice points corresponding to $R = 21.5\,\mathrm{nm}$. In both cases the energy levels are equally spaced and degeneracy follows the pattern 2, 4, 6, 8 as predicted by the analytical model.

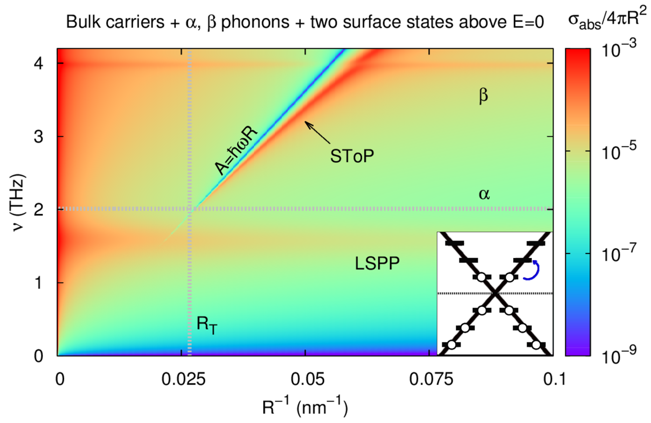

Supplementary Figure 2: Absorption cross-section of a TINP at finite temperature. For radii $R^{-1} < R_T^{-1} = (37.5\,\mathrm{nm})^{-1}$ (vertical dashed line) the effect of surface states is smeared by electron-phonon coupling. For small nanoparticles the effect remains at finite temperature due to the discrete nature of the surface states. Bulk dielectric function for this graph was measured at 300 K [7].

Supplementary Note 1 Topological insulator nanoparticle subject to light

The effect of a time-dependent electric field on the electrons in surface states can be analysed using time-dependent perturbation theory. Because the states decay quickly away from the surface we can set the variable $r = R$, the particle radius, everywhere. For simplicity let us for a moment consider a single surface state subject to circularly polarised light. Then the quasistatic potential of incident light gives rise to a potential energy of the electron with charge q in the surface state given by

$$q\Phi_{\text{in}}|_R = q\text{Re}[B_{\text{in}} R \sin\theta e^{i(\phi - wt)}] \tag{1}$$

The light wave is travelling along the z axis ($\mathbf{k}||z$) which coincides with the $\mathbf{c}$ axis of the material. This beam configuration was chosen because the azimuthal symmetry of the four-band Hamiltonian for the surface states allows the solution of the resultant equations analytically (the layered structure of Bi_2Se_3 crystal makes the in-plane dispersion different from that along c axis).

The resultant coupling between the surface states caused by external field leads to a surface charge density. To first order we have:

$$c_f^{(1)}(t) = -\frac{i}{\hbar}\int^t dt' \langle f| \Phi_{\rm in} |i\rangle e^{iw_{fi}t'} = -\frac{i}{\hbar}\int^t dt' \langle f| \frac{qB_{\rm in}R}{2}\sin\theta e^{i(\phi - wt')} + \frac{qB^*_{\rm in}R}{2}e^{-i(\phi-wt')} |i\rangle e^{iw_{fi}t'} =$$

$$= -\frac{\langle f| \frac{qB_{\rm in}R}{2}\sin\theta e^{i\phi}|i\rangle}{\hbar}\frac{e^{i(w_{fi}-w)t}}{w_{fi}-w} - \frac{\langle f| \frac{qB^*_{\rm in}R}{2}\sin\theta e^{-i\phi}|i\rangle}{\hbar}\frac{e^{i(w_{fi}+w)t}}{w_{fi}+w} \tag{2}$$

where $\hbar w_{fi} = E_f - E_i$. The lower integration limit is omitted because we assume a pure state $|i\rangle$ in the beginning (the perturbing potential can be slowly turned on from $t = -\infty$ if multiplied by $e^{\epsilon t}$ where ϵ is a small positive constant). The surface charge density arising from state $|i\rangle$ coupled to state $|f\rangle$ is given by

$$\sigma_{fi}(\theta,\phi,t) = c_f^{(1)}(t)\psi_i^\dagger(\theta,\phi)\psi_f(\theta,\phi)e^{-iw_{fi}t} + c.c. \tag{3}$$

To find the necessary matrix elements we use the surface states wavefunctions obtained by Imura *et al.* [1] (where wavefunction means the corresponding envelope function). The two-component wavefunctions satisfy the eigenvalue equation

$$\mathcal{H}\psi = \mathcal{H}\begin{pmatrix}\alpha_+\\ \alpha_-\end{pmatrix} = E\begin{pmatrix}\alpha_+\\ \alpha_-\end{pmatrix} \tag{4}$$

where $\mathcal{H}$ is the Dirac operator on the spherical surface of radius R given by [1]

$$\mathcal{H} = \begin{pmatrix} 0 & \frac{A}{R}(-\partial_\theta + \frac{i\partial_\phi}{\sin\theta} - \frac{\cot\theta}{2}) \\ \frac{A}{R}(\partial_\theta + \frac{i\partial_\phi}{\sin\theta} + \frac{\cot\theta}{2}) & 0 \end{pmatrix} \tag{5}$$

It is obtained from the low-energy four-band Hamiltonian of Bi_2Se_3 by projecting it onto two states with momentum along the surface $k_{||} = 0$ [1] (the value of $A = 3.0\,\mathrm{eV}\cdot\mathrm{Å}$ was obtained by averaging the relevant values over x, y and z directions from DFT calculations — see Table IV in [2]). The envelope function $(\alpha_+,\alpha_-)^T$ is an eigenstate and E — the corresponding eigenenergy. The eigenstates are labelled by three quantum numbers - $|s,n,m\rangle$. The "principal" quantum number $n = 0, 1, ...$ and azimuthal quantum number $m = 1/2, 3/2...$ (w.l.o.g. we focus on positive m onwards). The number $s = \pm$ is added for convenience — it denotes whether the state is below or above the Dirac point.

For a particular state $|s,n,m\rangle$ the solution has the form [1]:

$$\psi_{snm}(\theta,\phi) = \frac{e^{im\phi}}{\sqrt{4\pi R^2}}\begin{pmatrix}\alpha_{snm+}(\theta)\\ \alpha_{snm-}(\theta)\end{pmatrix} = \frac{e^{im\phi}}{\sqrt{4\pi R^2}}\begin{pmatrix}(1-cos\theta)^{\frac{1}{2}(m-\frac{1}{2})}(1+cos\theta)^{\frac{1}{2}(m+\frac{1}{2})}\beta_{nm+}(cos\theta)\\ -sgn(sm)(1-cos\theta)^{\frac{1}{2}(m+\frac{1}{2})}(1+cos\theta)^{\frac{1}{2}(m-\frac{1}{2})}\beta_{nm-}(cos\theta)\end{pmatrix} \tag{6}$$

where $\beta_{nm\sigma}$ are Jacobi polynomials:

$$\beta_{nm\sigma}(x) = c_{nm}J_n^{(m-\frac{\sigma}{2})(m+\frac{\sigma}{2})}(x) \tag{7}$$

with c_{nm} being the normalisation constant determined by

$$\int_{-1}^{1} dx(1-x)^\alpha(1+x)^\beta J_n^{\alpha\beta}J_{n'}^{\alpha\beta} = \delta_{nn'}\frac{2^{\alpha+\beta+1}(n+\alpha)!(n+\beta)!}{(2n+\alpha+\beta+1)(n+\alpha+\beta)!n!} = \delta_{nn'}\frac{1}{c_{nm}^2} \tag{8}$$

where $\alpha = m - \sigma/2$ and $\beta = m + \sigma/2$ in our case. The states are normalised over the surface of the particle and satisfy:

$$\langle s,n,m|s',n',m'\rangle = \delta_{s,s'}\delta_{n,n'}\delta_{m,m'} \tag{9}$$

Their energy is given by

$$E_{snm} = s\frac{A}{R}\left(n + |m| + \frac{1}{2}\right) \tag{10}$$

So the states are equally spaced with degeneracy increasing linearly in energy, i.e. 2, 4, 6, 8 etc. Let us now focus on a single state $|+,0,1/2\rangle$ for concreteness. The circularly polarised light (Eq. (1)) has $m_z = 1$ and couples this state to other ones:

$$\langle +, 0, 3/2 | \sin\theta e^{i\phi} | +, 0, 1/2\rangle = \sqrt{\frac{2}{3}} \tag{11}$$

$$\langle +, 1, -1/2 | \sin\theta e^{-i\phi} | +, 0, 1/2\rangle = \frac{\sqrt{2}}{3} \tag{12}$$

$$\langle -, 0, 1/2 | \sin\theta e^{-i\phi} | +, 0, 1/2\rangle = \frac{2}{3} \tag{13}$$

Together with Eq. (3) and (6) they allow to find the time-dependent surface charge density given by

$$\sigma(\theta,\phi,t) = 2q\mathrm{Re}\left[-\frac{qB_{\mathrm{in}}R\sin\theta e^{i(\phi-wt)}}{8\pi R^2(E_{+0\,3/2}-E_{+0\,1/2}-\hbar w)} - \frac{qB^*_{\mathrm{in}}R\sin\theta e^{-i(\phi-wt)}}{24\pi R^2(E_{+1\,-1/2}-E_{+0\,1/2}+\hbar w)} - \frac{qB^*_{\mathrm{in}}R\sin\theta e^{-i(\phi-wt)}}{12\pi R^2(E_{-0\,-1/2}-E_{+0\,1/2}+\hbar w)}\right]$$

$$= -q^2\left(\frac{1}{4\pi(A-\hbar wR)} + \frac{1}{12\pi(A+\hbar wR)} + \frac{1}{6\pi(-2A+\hbar wR)}\right)\mathrm{Re}[B_{\mathrm{in}}\sin\theta e^{i(\phi-wt)}] \tag{14}$$

The divergence of the first term signifies an absorption of a photon that causes the transition $|+, 0, 1/2\rangle \rightarrow |+, 0, 3/2\rangle$. On the other hand, the resonance of the last term corresponds to transition $|+, 0, 1/2\rangle \rightarrow |-, 0, -1/2\rangle$ during which a photon is emitted. In fact, we should omit the last transition because we assumed that all states below are filled. Then we can write off-resonant surface charge density in terms of potential inside the particle as

$$\sigma(\mathbf{r},t) = \frac{-\delta^+_R\epsilon_0\Phi_{\mathrm{in}}|_R}{R} = -\delta^+_R\epsilon_0\mathrm{Re}[B_{\mathrm{in}}\sin\theta e^{i(\phi-wt)}] \tag{15}$$

where the superscript denotes $sgn(m_z)$ of the potential (1) and

$$\delta^+_R(w) = \frac{q^2}{\epsilon_0}\left(\frac{1}{4\pi(A-\hbar wR)} + \frac{1}{12\pi(A+\hbar wR)}\right) \tag{16}$$

The potential outside the TINP is given by

$$\Phi_{\mathrm{out}} = \mathrm{Re}\left[\frac{C_{\mathrm{out}}}{r^2}\sin\theta e^{i\phi} - \mathcal{E}r\sin\theta e^{i\phi}\right] \tag{17}$$

where $\mathcal{E}$ is the incident field strength. Φ_{out} should now be matched to Φ_{in} using the boundary conditions on the electric field where the surface charge density σ (and δ^+_R) now enters:

$$\frac{\partial\Phi_{\mathrm{in}}}{\partial\theta}|_R = \frac{\partial\Phi_{\mathrm{out}}}{\partial\theta}|_R \tag{18}$$

$$\epsilon_{\mathrm{in}}\frac{\partial\Phi_{\mathrm{in}}}{\partial r}|_R = \frac{\partial\Phi_{\mathrm{out}}}{\partial r}|_R + \sigma \tag{19}$$

Solving these equations we obtain

$$B_{\mathrm{in}} = \frac{-3}{\epsilon_{in} + \delta^+_R + 2}\mathcal{E} \tag{20}$$

$$C_{\mathrm{out}} = R^3\frac{\epsilon_{\mathrm{in}} + \delta^+_R - 1}{\epsilon_{\mathrm{in}} + \delta^+_R + 2}\mathcal{E} \tag{21}$$

The latter relates the induced dipole moment to the external field allowing to find the absorption cross section. The contribution of the external field to spin-orbit coupling is negligible and was not considered above. The perturbation theory is valid for $qB_{\mathrm{in}}R \ll \frac{A}{R}$ which translates to $\mathcal{E}$ values below $\sim 10^6$ V/m for $R = 5$ nm.

Alternatively we could have used potential of incident light given by

$$\Phi_{\mathrm{in}} = q\mathrm{Re}[B_{\mathrm{in}}r\sin\theta e^{i(\phi+wt)}] \tag{22}$$

with azimuthal component of angular momentum $m_z = -1$. The net effect on the response of the particle is to change the sign in front of w in the δ_R^+ term which should now be substituted in Eqs. (15), (20) and (21) by

$$\delta_R^- = \frac{q^2}{\epsilon_0}\left(\frac{1}{4\pi(A+\hbar wR)} + \frac{1}{12\pi(A-\hbar wR)}\right) \tag{23}$$

where only the middle term can be resonant. Thus a single surface state reacts differently to different circular polarisations because of intrinsic anisotropy — the states have half-integer m.

Let the Fermi energy equal A/R so that both states $|+,0,\pm1/2\rangle$ are occupied. From calculating matrix elements it then turns out that only these two states can couple to other states under the influence of light. Effect of all the occupied states below can be neglected because they have transition energies of at least $4A/R$ (e.g. $|-,1,\pm1/2\rangle \to |+,0,\pm3/2\rangle$). The resultant surface charge density due to the states $|+,0,\pm1/2\rangle$ will have the same symmetry as the incident light. In all cases ($m_z = \pm1$, x-, y-polarised light) we have

$$\delta_R = \frac{q^2}{\epsilon_0}\left(\frac{1}{4\pi(A+\hbar wR)} + \frac{1}{12\pi(A-\hbar wR)} + \frac{1}{4\pi(A-\hbar wR)} + \frac{1}{12\pi(A+\hbar wR)}\right) \tag{24}$$

On the other hand, if the Fermi energy was located at $-A/R$ only the states $|-,0,\pm1/2\rangle$ would undergo transitions (again neglecting the transitions with energy $4A/R$ and higher) in which case we have (as follows from the last term in Eq. (14))

$$\delta_R = \frac{q^2}{\epsilon_0}\left(\frac{1}{6\pi(2A-\hbar wR)} + \frac{1}{6\pi(2A+\hbar wR)}\right) \tag{25}$$

To summarise the above, an external potential applied to an initially neutral nanoparticle enters the Hamiltonian giving rise to a surface charge density. This surface charge density also enters the Hamiltonian making the problem self-consistent in nature. The initial state we chose ($|\pm,0,\pm1/2\rangle$) are special because the resultant surface charge density has the same angular dependence (dipolar) as the external potential allowing to solve the equations for the electric field analytically. In general, surface states give rise to a surface charge density which is a linear combination of spherical harmonics which in turn creates higher harmonics in the potential.

Supplementary Note 2 Tight-binding calculation of surface spectrum

Our calculations are based on the model Imura et al [1] who used the four-band low-energy Hamiltonian to obtain tight-binding Hamiltonian using the substitutions:

$$k_i \to \frac{1}{L}\sin k_i L \tag{26}$$

$$k_i^2 \to \frac{2}{L^2}(1-\cos k_i L) \tag{27}$$

where $i = x, y, z$. This yields the following Hamiltonian:

$$H = \sum_k [\tau_z(m_0 + \frac{2}{L^2}\sum_{i=x,y,z} m_2(1-\cos k_i L)) + \frac{1}{L}A\tau_x \sum_{i=x,y,z}\sigma_i \sin k_i L]c_k^\dagger c_k \tag{28}$$

The Hamiltonian above can be Fourier transformed yielding a nearest neighbour model on cubic lattice in real space:

$$H = \sum_{lmn}(m_0 + \frac{6}{L^2}m_2)\tau_z c_{lmn}^\dagger c_{lmn} + \sum_{lmn}[(-\frac{1}{L^2}m_2\tau_z - \frac{i}{2L}A\tau_x\sigma_x)c_{l+1mn}^\dagger c_{lmn} + (m+1), (n+1)\text{ terms} + \text{h.c.}] \tag{29}$$

For the Hamiltonian above we use values for Bi_2Se_3 from DFT calculations averaged over x, y and z directions [2]: $m_0 = -0.28$ eV, $A = 3.0\,\text{eV}\cdot\text{Å}$, $m_2 = 32\,\text{eV}\cdot\text{Å}^2$. The decay constant of surface states into the bulk with these parameters is given by Eq. 15 in [1]:

$$Re[\kappa] = Re\left[\frac{A \pm \sqrt{A^2 + 4m_0 m_2}}{2m_2}\right] \approx 0.5\,\text{nm}^{-1} \tag{30}$$

The only 'fitting' parameter for the tight-binding model is the length between the lattice points, L. We take $L = 36\,\text{Å}$ in accordance with the repeat length of Bi_2Se_3 hexagonal cell along c-axis (28.64 Å). Thus our coarse grained model consists of cubic cells each representing several primitive unit cells of the material. This is justified because we are only interested in delocalised surface states lying low in energy. Examples of energy spectra close to the Dirac point for cubic and spherical shapes are shown in Supplementary Figure 1. The spectrum is symmetric around the Dirac point due to the particle hole symmetry inherent in the initial model Hamiltonian [1]. The states closest to the Dirac point are in best agreement between the analytical and tight-binding models. The deviations further from the Dirac point occur due to the small sizes of the particles than are computationally attainable. Nevertheless, the energy spacing between levels is constant and degeneracy follows the pattern 2, 4, 6, 8... as predicted by the analytical model in Eq. 10. Similarity of spectra for cubic and spherical shapes highlights the topological nature of the surface states.

Supplementary Note 3 Finite temperature simulations

As mentioned in the article, early studies found that the surface states couple to the surface phonon developed from bulk α [3, 4]. The coupling to a single optical phonon at ~ 8 meV was later confirmed with time-resolved ARPES [5] as well as transport measurements [6]. To model the effect of finite temperature we assume that in nanoparticles with the level spacing of < 8 meV the effect of the surface states is completely destroyed by coupling to the phonons. Furthermore, we assume that the acoustic phonons that lie lower in energy can also contribute to the scattering. This is a strict approximation because it does not account for energy and momentum conservation that restrict the scattering between the few discrete states available. Finite temperature enters our model by multiplying the δ_R term due to the surface states with a smooth step function centered at $R_T = 37.5\,\text{nm}$ (corresponds to surface level spacing of $A/R_T = 8$ meV). For finite temperature simulations we use ϵ_{in} measured at 300 K on a different Bi_2Se_3 sample [7]. The resulting absorption cross section presented in Supplementary Figure 2 is valid for $T \leq 40$ K (temperature of experiments [5, 6]) but may also hold at higher temperature due to the considerations outlined in the article.

Supplementary References

[1] Ken-Ichiro Imura, Yukinori Yoshimura, Yositake Takane, and Takahiro Fukui. Spherical topological insulator. *Phys. Rev. B* 86, 235119 (2012).

[2] Chao-Xing Liu, Xiao-Liang Qi, HaiJun Zhang, Xi Dai, Zhong Fang, and Shou-Cheng Zhang. Model Hamiltonian for topological insulators. *Phys. Rev. B* 82, 045122 (2010).

[3] Xuetao Zhu, L. Santos, R. Sankar, S. Chikara, C. . Howard, F. C. Chou, C. Chamon, and M. El-Batanouny. Interaction of Phonons and Dirac Fermions on the Surface of Bi_2Se_3: A Strong Kohn Anomaly. *Phys. Rev. Lett.* 107, 186102 (2011).

[4] Xuetao Zhu, L. Santos, C. Howard, R. Sankar, F. C. Chou, C. Chamon, and M. El-Batanouny. Electron-Phonon Coupling on the Surface of the Topological Insulator Bi_2Se_3 Determined from Surface-Phonon Dispersion Measurements. *Physical Review Letters* 108, 185501 (2012).

[5] J. A. Sobota, S.-L. Yang, D. Leuenberger, A. F. Kemper, J. G. Analytis, I. R. Fisher, P. S. Kirchmann, T. P. Devereaux, and Z.-X. Shen. Distinguishing bulk and surface electron-phonon coupling in the topological insulator Bi_2Se_3 using time-resolved photoemission spectroscopy. *Phys. Rev. Lett.* 113, 157401 (2014).

[6] M. V. Costache, I. Neumann, J. F. Sierra, V. Marinova, M. M. Gospodinov, S. Roche, and S. O. Valenzuela. Fingerprints of inelastic transport at the surface of the topological insulator Bi_2Se_3: Role of electron-phonon coupling. *Phys. Rev. Lett.* 112, 086601 (2014).

[7] P. Di Pietro, M. Ortolani, O. Limaj, A. Di Gaspare, V. Giliberti, F. Giorgianni, M. Brahlek, N. Bansal, N. Koirala, S. Oh, P. Calvani, and S. Lupi. Observation of Dirac plasmons in a topological insulator. *Nature Nanotechnology* 8, 556–560 (2013).
